# Non-small Cell Lung Carcinoma With Metastases to Various Levels of Vertebra: A Case Report and Literature Review

**DOI:** 10.7759/cureus.37851

**Published:** 2023-04-19

**Authors:** Pugazhendi Inban, Tamer Zahdeh, Sai Harini Chandrasekaran, Tarsha A Intsiful, Chengala Ananyaa Gowthavaram, Abasiono K Ebong, Yabets Kejela, Satyam Singh, Aadil Khan, Ayushi Awale

**Affiliations:** 1 Department of General Medicine, Government Medical College, Omandurar, Chennai, IND; 2 Internal Medicine, Hadassah Medical Center, Jerusalem, ISR; 3 College of Medicine, University of Ghana Medical Center, Accra, GHA; 4 Internal Medicine, Malla Reddy Institute of Medical Sciences, Hyderabad, IND; 5 College of Medicine, Richmond Gabriel University, Arnos Vale, VCT; 6 Department of Surgery, Hayat Hospital Medical College, Addis Ababa, ETH; 7 Internal Medicine, Ganesh Shankar Vidyarthi Memorial (GSVM) Medical College, Kanpur, IND; 8 Department of Internal Medicine, Lala Lajpat Rai (LLR) Hospital, Kanpur, IND; 9 Internal Medicine, Star Hospital, Lalitpur, NPL

**Keywords:** chemotherapy, pet scans, vertebra fracture, spinal paraplegia, metastatic non-small cell lung cancer

## Abstract

The consequences of lung cancer over the past century have been quite deadly and cost millions of lives. Besides the statistics that show its brutal mortality rate, the comorbidities secondary to lung cancer have had their toll and burden on patients too. Lung cancer is broadly and histologically divided into small and non-small cell lung cancers (NSCLC), with the latter associated with a heavy smoking history. Initial presentation of NSCLC varies, and many patients present with an advanced disease that has spread to different parts of the body. Metastasis to the bone can lead to severe pain requiring extreme analgesia regimens. Here, we present a case of a 68-year-old male with advanced NSCLC who initially presented with bony pain due to metastasis.

## Introduction

Lung cancer remains one of the leading causes of morbidity and mortality in the United States, with non-small cell lung cancer (NSCLC) accounting for most cases [1]. Smoking is still the leading risk factor, making NSCLC one of the most preventable cancers because of early findings using annual low-dose computed tomography (CT) scans [2]. Many patients have advanced disease at presentation, and symptoms vary depending on the extent of cancer spread, from local symptoms such as cough and hemoptysis to distant ones arising from metastasis. Bone, particularly the vertebral column, is one of the most common places where NSCLC frequently metastasizes. Approximately 20-30% of patients with non-small cell lung cancer (NSCLC) present with bone metastases at diagnosis, and 35-60% will develop them during their disease course. Vertebral metastasis is a common complication of lung cancer, and it occurs in 30-40% of patients with advanced disease. NSCLC bone metastasis appears as osteolytic lesions on radiographs, and almost all patients complain of aching pain that is worse on body movements and awakes them from sleep [3]. Managing bony metastasis symptoms in NSCLC involves a holistic and multimodal approach to pain in patients with bone metastases which include chemotherapy, radiotherapy, bisphosphonates, opioids, and other avenues. The decision to use any previous interventions depends on the extent of metastasis and severity of symptoms, with bony metastases responding well to radiotherapy. Generally, patients have better outcomes which depend on gender, early diagnosis, adenocarcinoma, solitary bone metastasis, no metastasis to the appendicular bone, no pathologic fracture [4]. Surgery might even be required in patients with extensive bony metastasis, especially those at high risk for fractures or spinal cord compression, which usually indicates a poorer prognosis [5].

## Case presentation

A 68-year-old male with a known case of hypertension was brought to the emergency department with sudden onset numbness in the lower half of the body, followed by walking difficulty for the last six hours. He also complained of progressive back pain for the last six months, worsening and relieved by analgesics from the local physician. He was compliant with his medications and had an extensive history of smoking with 30 pack-year, which he quit two years back. He had a history of dry cough with minimal hemoptysis two weeks ago and non-documented weight loss. He denied any history of night sweats and recurrent lung infections.

**Figure 1:**
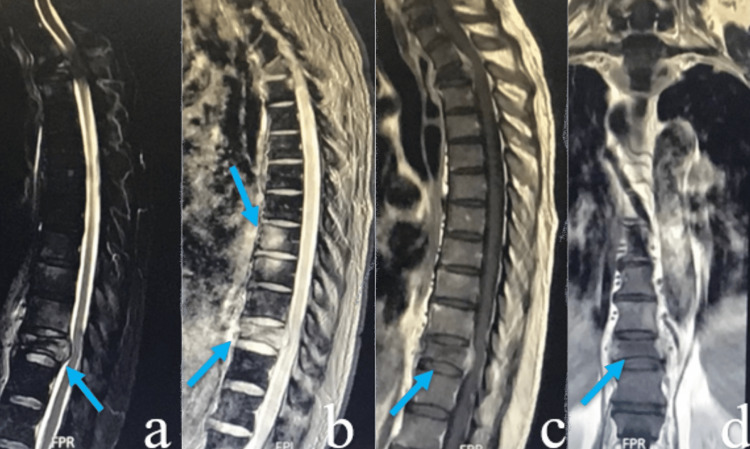
MRI demonstrating pathological collapse fracture in short tau inversion recovery (STIR) weighted (a), T1-weighted (b), T2-weighted (c, d) images of T2 and T11 vertebrae.

**Figure 2:**
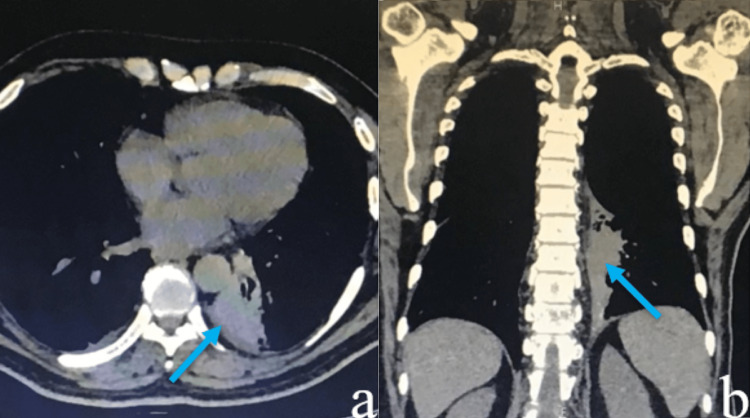
CT demonstrating the left hilum involving the left lung bronchus (a), with the destruction of dorsal vertebrae (b).

On physical exam, he was hemodynamically stable and oriented to time, place, and person. Tendon reflexes and pain sensitivity were preserved, and the power was 4/5 in both lower limbs. Chest x-ray revealed hyperinflated lungs with a discrete central infiltrate. Magnetic resonance imaging (MRI) lumbosacral spine showed patchy areas of altered signal intensities seen in multiple dorsal and lumbar vertebral bodies showing a hyperintense signal on T2 and a hypointense signal on T1WI. A partial T2 and T11 vertebrae collapse with retropulsion fragments and epidural soft tissue causing compression over the spinal cord was also noticed. Additionally, a paravertebral soft tissue at T1-2 and T10-11 levels showed enhancement on a postcontrast scan, indicating metastasis, as shown in Figure 1. A whole-body CT scan was done for further investigation to find a possible primary locus of carcinoma. An ill-defined soft tissue was found at the left hilum involving the left lung bronchus with the destruction of dorsal vertebrae, as shown in Figure 2.

The patient was then suspected of having lung malignancy and was kept under supportive care and high observation. A left lung bronchoscopy-guided biopsy was performed. Biopsy specimens were examined and revealed tissue bits showing alveolar spaces lined by hyperplastic and moderately pleomorphic columnar cells having round to oval hyperchromatic nuclei and a scant to moderate amount of eosinophilic cytoplasm at places infiltrating the subepithelial tissue. Mixed inflammatory infiltrates were seen too, which confirmed the diagnosis of NSCLC. On further tumor screening, he was positive for programmed death ligand-1 (PD-L1) and endothelial growth factor receptor (EGFR). Consequently, the patient was managed by the combination of cisplatin (50 mg/m^2^), epirubicin (60 mg/m^2^), cyclophosphamide (600 mg/m^2^) (PEC regimen), given every four weeks, and pembrolizumab (a monoclonal antibody) to enhance immunity to fight cancer. His pain was managed with combination analgesia.

## Discussion

**Figure 3:**
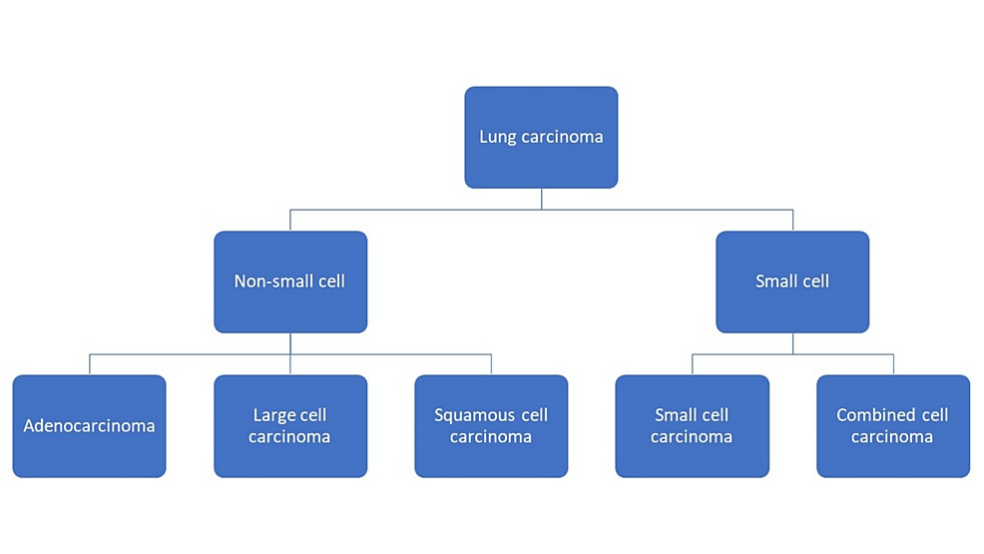
Lung carcinoma and its subtypes.

Lung cancer, by far, is considered the leading cause of cancer death. It is estimated that there are about 350 deaths every day due to lung malignancy in the United States [6]. While smoking remains the major risk factor for lung cancer, other factors can also increase the risk, such as occupational hazards like asbestos exposure, which has a synergistic effect with cigarette smoking in raising the risk for lung cancer. Radon, secondhand smoking, certain dietary habits, consuming alcohol, air pollution, and many more also contribute to developing lung malignancy [7]. Patients present with various symptoms like hemoptysis, shortness of breath, or recurrent localized pneumonia. Other symptoms might also be present depending on the type of lung cancer, which is broadly divided into small-cell that can be accompanied by paraneoplastic syndromes, and NSCLC, which is further divided into more subtypes, most notably squamous cell carcinoma (Figure 3). Some patients with NSCLC can be asymptomatic until later in the disease when they have a well-advanced presentation, mainly secondary to metastasis to other body parts [8]. Bony metastasis is one of the most common complications of unchecked lung malignancy. Bony pain is the hallmark chief complaint, and radiographs frequently show osteolytic lesions.

Vertebral metastasis is a common complication of lung cancer, and it occurs in 30-40% of patients with advanced disease [9]. The most common sites of vertebral metastasis are the thoracic and lumbar spine. The metastatic cells can invade the bone tissue and cause bone destruction, which can lead to pain, spinal cord compression, and pathological fractures [2]. The mechanism of bone destruction involves the activation of osteoclasts, which are cells that break down bone tissue. Several factors mediate osteoclast activation, including cytokines, growth, and tumor-derived factors. The clinical presentation of lung cancer with vertebral metastasis depends on the location and extent of the metastasis [10]. The most common symptom is pain, which can be localized or diffuse and can be exacerbated by movement or pressure. The pain can be severe and interfere with daily activities [11]. Spinal cord compression can cause neurological deficits, such as weakness, numbness, and bowel or bladder dysfunction. Pathological fractures can cause deformity and loss of mobility. Other symptoms include weight loss, fatigue, and cough [4].

Managing lung cancer with vertebral metastasis depends on several factors, extent of the metastasis, the location of the metastasis, the histology of cancer, and the patient's overall health status [12]. Treatment options include surgery, radiotherapy, chemotherapy, targeted therapy, pain management, and immunotherapy. Managing NSCLC can be complicated but depends primarily on the stage of disease, along with other comorbidities patients might have. Stage 0 and I usually respond well to endobronchial procedures and surgery, respectively, considering that the patient is eligible for either one of the options [8]. Stage II and beyond require more extreme measures involving pneumonectomy, chemotherapy, and radiotherapy [13]. In bony metastasis, pain is a significant issue, and many patients might require opioids to relieve their symptoms. Different chemotherapeutic regimens are used in advanced NSCLC, with cisplatin being notoriously known to be effective in managing NSCLC side by side with radiotherapy [14]. After establishing the diagnosis, our patient was commenced on the PEC regimen: a combination of cisplatin (50 mg/m^2^), epirubicin (60 mg/m^2^), and cyclophosphamide (600 mg/m^2^) which were given every four weeks along with pembrolizumab, a monoclonal antibody, to enhance cancer-fighting immunity. The patient responded well to the treatment given and continued to follow up.

## Conclusions

Lung cancer is the leading cause of cancer mortality. The presentation of lung cancer can widely vary from local symptoms to those that result from distant spreading. Bony pain in a patient with a heavy smoking history should always raise the suspicion of subtle lung malignancy, such as the case in our patient. Further workup, including tissue biopsy and imaging, is crucial to determine the type and stage of lung cancer. Managing bony metastasis symptoms in NSCLC involves a holistic and multimodal approach, involving chemotherapy, radiotherapy, bisphosphonates, opioids, and other avenues to improve the quality of life.
